# Acknowledgement to Referees

**DOI:** 10.1007/s40801-016-0095-0

**Published:** 2016-10-31

Dear Reader,

As we approach the end of 2016, and publish our final issue of *Drugs—Real World Outcomes* for the year, we wish to reflect on another successful year for the journal and for others in the Adis Premier journals portfolio, and to thank all who have contributed to *Drugs—Real World Outcomes* over the past 12 months.

An important milestone was reached for *Drugs—Real World Outcomes* in that the journal content is now available on PubMed Central. Another of our open access journals, *Drug Safety—Case Reports,* is also now available on PubMed Central.

As of September 2016, *Drugs—Real World Outcomes’* top 10 downloaded articles from SpringerLink this year were:

- Elliott, R.A., Lee, C.Y., Beanland, C. et al. Medicines Management, Medication Errors and Adverse Medication Events in Older People Referred to a Community Nursing Service: A Retrospective Observational Study. Drugs—Real World Outcomes (2016) 3: 13.
- Kocis, P.T., Liu, G., Makenbaeva, D. et al. Use of Chronic Medications Among Patients with Non-Valvular Atrial Fibrillation. Drugs—Real World Outcomes (2016) 3: 165.
- Mast, G., Fernandes, K., Tadrous, M. et al. Persistence of Antipsychotic Treatment in Elderly Dementia Patients: A Retrospective, Population-Based Cohort Study. Drugs—Real World Outcomes (2016) 3: 175.
- Lajara, R., Nikkel, C. & Abbott, S. The Clinical and Economic Impact of the V-Go^®^ Disposable Insulin Delivery Device for Insulin Delivery in Patients with Poorly Controlled Diabetes at High Risk. Drugs—Real World Outcomes (2016) 3: 191.
- Watras, M.M., Patel, J.P. & Arya, R. Traditional Anticoagulants and Hair Loss: A Role for Direct Oral Anticoagulants? A Review of the Literature. Drugs—Real World Outcomes (2016) 3: 1.
- Mold, J.W. & Holtzclaw, B.J. Selective Serotonin Reuptake Inhibitors and Night Sweats in a Primary Care Population. Drugs—Real World Outcomes (2015) 2: 29.
- Aggarwal, N. Drug-Induced Subacute Cutaneous Lupus Erythematosus Associated with Proton Pump Inhibitors. Drugs—Real World Outcomes (2016) 3: 145.
- Rojas-Fernandez, C.H. Can 5-HT_3_ Antagonists Really Contribute to Serotonin Toxicity? A Call for Clarity and Pharmacological Law and Order. Drugs—Real World Outcomes (2014) 1: 3.
- Muduma, G., Odeyemi, I. & Pollock, R.F. Evaluating the Cost-Effectiveness of Prolonged-Release Tacrolimus Relative to Immediate-Release Tacrolimus in Liver Transplant Patients Based on Data from Routine Clinical Practice. Drugs—Real World Outcomes (2016) 3: 61.
- de Jong, J., Garne, E., de Jong-van den Berg, L.T.W. et al. The Risk of Specific Congenital Anomalies in Relation to Newer Antiepileptic Drugs: A Literature Review. Drugs—Real World Outcomes (2016) 3: 131.

We offer our sincere thanks to all authors who have contributed articles to *Drugs—Real World Outcomes* over the course of 2016. Their skill and dedication are critical to the continued publication of the journal. The quality of published articles is, similarly, testament to the significant efforts of the peer reviewers, whose commitment ensures that the journal’s content is held to the highest possible standard. We would like to thank the following individuals who acted as reviewers for *Drugs—Real World Outcomes* in the last 12 months:

*Adebowale Dele Ademola*, Nigeria

*Kraemer Alwin*, Germany

*Richard H. Aster*, USA

*Pierrick Bedouch*, France

*Nancy Bernardy*, USA

*Benoit Boland*, Belgium

*Machaon Bonafede*, USA

*Neil Brickel*, UK

*Marta Brooks*, USA

*Anette Bygum*, Denmark

*Johanna Callhoff*, Germany

*Wendy Cheng*, USA

*Victoria Collings*, UK

*Rohan A. Elliott*, Australia

*Barbara Farrell*, Canada

*Paula Fresco*, Portugal

*Masahiro M. Fukuoka*, Japan

*Chris Gillette*, USA

*B. Joseph Guglielmo*, USA

*Zeky Yüksel Gunaydin*, Turkey

*Mark Haggard*, UK

*Jesper Hallas*, Denmark

*Mainul Haque*, Malaysia

*Swapnil Hiremath*, Canada

*Keith B. Hoffman*, USA

*Chun-Ta Huang*, Taiwan

*Klejda Hudhra*, Spain

*Sekwon Jang*, USA

*Susan Jick*, USA

*Pravin Kamble*, USA

*Harparkash Kaur*, UK

*Donald G. Klepser*, USA

*Paul T. Kocis*, USA

*Sam Kosari*, Australia

*Richard Laing*, USA

*Cecilie Johannessen Landmark*, Norway

*Marianne Lisby*, Denmark

*Eleanor Lucas*, USA

*Hendrika Luijendijk*, The Netherlands

*Anke H. Maitland-van der Zee*, The Netherlands

*Louise Mallet*, Canada

*G. B. John Mancini*, Canada

*Gloria Manso*, Spain

*John F. McCarthy*, USA

*Natalie McCormick*, Canada

*Anthony B. Miller*, Canada

*Andrew Mosholder*, USA

*Domenico Motola*, Italy

*Marco Mula*, UK

*Giorgio Mustacchi*, Italy

*Paul N. Newton*, UK

*Onyema Ogbuagu*, USA

*Jan Palmblad*, Sweden

*Jean-Jacques Parienti*, France

*Timo Partonen*, Finland

*Kathryn Peri*, New Zealand

*Sathirakorn Pongpanich*, Thailand

*Valentin Prieto-Centurion*, USA

*Neal Ready*, USA

*Jenna M. Reps*, UK

*John Robst*, USA

*Albert Roger*, Spain

*Josea Rono*, Kenya

*Sabine Ruths*, Norway

*Alan Schorr*, USA

*Todd Semla*, USA

*Pasquale Striano*, Italy

*Roger E. Thomas*, Canada

*Philippe Thuillier*, France

*Petra A. Thurmann*, Germany

*James E. Tisdale*, USA

*Giuliano Tocci*, Italy

*Antonella Tosti*, USA

*Robert Vander Stichele*, Belgium

*Bastiaan Venhuis*, The Netherlands

*Pa-Chun Wang*, Taiwan

*Juanita Westbury*, Australia

*Peter K.K. Wong*, Australia

*Antoinette A. Wozniak*, USA

In addition, we would like to thank the members of the journal’s Honorary Editorial Board, who have acted as peer reviewers and authors, and have provided guidance on journal content, policy and processes:

*G.C. Alexander*, Johns Hopkins University, Baltimore, MD, USA

*A. Beresniak*, Data Mining International, Geneva, Switzerland

*M.L. Berger*, Pfizer, Inc., New York, NY, USA

*H. Birnbaum*, Analysis Group, Inc., Boston, MA, USA

*J.M. Bottomley*, Amygdala Ltd., Letchworth Garden City, England

*P. Denig*, University of Groningen, Groningen, The Netherlands

*J.E. Fincham*, Presbyterian College School of Pharmacy, Clinton, SC, USA

*B. Godman*, Karolinska Institutet, Stockholm, Sweden

*D. Goldsmith*, Goldsmith Pharmacovigilance and Systems, New York, NY, USA

*S. Karve*, AstraZeneca, Gaithersburg, MD, USA

*N.Y. Kirson*, Analysis Group, Inc., Boston, MA, USA

*C. Kozma*, CK Consulting Associates, St. Helena Island, SC, USA

*T. Lasky*, MIE Resources, Baltimore, MD, USA

*A.A. Mangoni*, Flinders University, Adelaide, SA, Australia

*S. Mimica-Matanovic*, University Hospital Osijek, Osijek, Croatia

*A. Modi*, Merck, Whitehouse Station, NJ, USA

*J.K. Sluggett*, Monash University, Parkville, VIC, Australia

*K.A. Swanson*, University of Oklahoma, Oklahoma City, OK, USA

*J.E Ware Jr*., University of Massachusetts, Worcester, MA, USA

*Q. Zou*, The Lewin Group, Falls Church, VA, USA

In other portfolio news, mid-2016 saw the release of the ISI Journal Citation Reports and the latest journal impact factors. The majority of our journals saw a rise in impact factor against the previous year. Most notably, five of our journals saw an increase of over 10% in impact factor – *The Patient* (17.08*%), Clinical Drug Investigation* (15.99%), the *American Journal of Clinical Dermatology* (13.17%), *Drug Safety* (13.5%), and *Drugs* (12.43%).

The Adis journals portfolio will expand in 2017, with the launch of *PharmacoEconomics—Open*. This new journal will be fully open access and will focus on the publication of applied research on the economic implications and health outcomes associated with drugs, devices and other healthcare interventions. It joins our other well respected health outcomes publications, *PharmacoEconomics, Applied Health Economics and Health Policy,* and *The Patient: Patient-Centered Outcomes Research*.

We hope that you have found the articles published throughout 2016 to be both interesting and informative. We have appreciated the high quality of content contributed to the journal this year and look forward to keeping you up to date with topical real-world outcomes in 2017.

With best wishes from Dene Peters (Editor-in-Chief) and Kathy Fraser (Deputy Editor).

